# Supplementary material for: Nutritional status and treatment outcomes of tuberculosis in Mizan Tepi University Teaching Hospital, a five -year retrospective study
Source: PLoS One. 2024 Feb 15;19(2):e0298244. doi: 10.1371/journal.pone.0298244 (PMC10868852; doi:10.1371/journal.pone.0298244)
Supplement: S2 File — (DOCX) [file pone.0298244.s002.docx]

**Checklist for data extraction for the research “ nutritional status and Treatment Outcomes of Tuberculosis in Mizan Tepi University Teaching Hospital, A five -year Retrospective Study ( from January 1, 2016, to December 31, 2020)**

| Code | Sex | Age | Residence | Weight (Kg) | Height (meter) | BMI (Kg/m^2^) | Type of TB (PTB, EPTB) | Smear result (N, P) | HIV status (N, P) | Category of TB  (NC, R, F, C) | Year of TB treatment (2016- 2020) | Anti-TB treatment outcome  (C, TC, F, Df, D) |  |  |
| --- | --- | --- | --- | --- | --- | --- | --- | --- | --- | --- | --- | --- | --- | --- |
| 001 |  |  |  |  |  |  |  |  |  |  |  |  |  |  |
| 002 |  |  |  |  |  |  |  |  |  |  |  |  |  |  |
| 003 |  |  |  |  |  |  |  |  |  |  |  |  |  |  |
| 004 |  |  |  |  |  |  |  |  |  |  |  |  |  |  |
| 005 |  |  |  |  |  |  |  |  |  |  |  |  |  |  |
| 006 |  |  |  |  |  |  |  |  |  |  |  |  |  |  |

**PTB= pulmonary TB, EP=extra-pulmonary, N=negative, P=positive, NC=new case, R=relapse, C=cured, TC=treatment completed, F=failure, DF=default, D=death.**

Name of data collector________________________

Sign of data collector _________________________

Date of data collection ________________________
