# Supplementary material for: Nutritional status and treatment outcomes of tuberculosis in Mizan Tepi University Teaching Hospital, a five -year retrospective study
Source: PLoS One. 2024 Feb 15;19(2):e0298244. doi: 10.1371/journal.pone.0298244 (PMC10868852; doi:10.1371/journal.pone.0298244)
Supplement: Attachment — Submitted filename: Reviewers comments.docx [file pone.0298244.s003.docx]

Research title: Nutritional Status and Treatment Outcomes of Tuberculosis in Mizan Tepi University Teaching Hospital - A five year Retrospective Study

**General comment**

The research title is highly relevant and scientifically significant. However, the research design and the approach to conducting the study may not be appropriate for effectively addressing the research questions. Additionally, the manuscript's writing style appears outdated and does not align with modern standards. Significant revisions are required for the manuscript. Furthermore, the English writing in the manuscript could benefit from substantial improvement.

**Abstract**

1. Background: The research gap that the current study intends to address is not well described. The researcher states that there is limited information regarding the impact of nutritional status on TB treatment outcomes. However, there have been studies investigating the association between nutritional status and TB treatment outcomes. I suggest revising the background section to focus on the relationship between nutritional status and TB treatment outcomes, clearly highlighting the existing research gaps.

2. Method: The study design employed by the researchers is not clearly articulated. Was it a retrospective cohort study? If it was indeed a retrospective cohort study, they need to define the exposure and the outcome. Additionally, they should describe the timing of nutritional status assessment and treatment outcomes. Was the nutritional status evaluated at the initiation of treatment, after the second month of treatment, or after six months of treatment?

3. Results: The statement "A total of 625 TB patients with complete information were included from the registration book" could be relocated to the methods section. The results presented in the abstract should be aligned with the main objective of the research. Other findings may be included in the results section if they are deemed pertinent. This section requires further revision.

4. Conclusion: The conclusion section also needs to be aligned with the research objectives and major findings. The primary aim of the research was not to determine the magnitude of treatment success.

**Introduction**

1. It is advisable to merge the first and second paragraphs. Summarizing the burden of TB and its related mortality in a single paragraph would be more effective. This approach would allow the researchers to discuss the relationship between nutritional status and TB treatment outcomes more comprehensively within the introduction section.

2. Other factors associated with TB treatment outcomes fall beyond the scope of this research, given the misalignment between the researchers' title and objective.

3. The researchers have omitted mention of existing studies investigating the association between nutritional status and TB treatment in Ethiopia within the introduction section. While they have referenced other studies focusing on factors affecting TB treatment outcomes in Ethiopia, this omission suggests a bias in that section.

**Methods**

1. Study area and period: Some of the information provided appears unnecessary for the study. The description of the setting is crucial for replication and generalization. It's advisable to include only pertinent and relevant information. For instance, the statement "The hospital has antenatal care (ANC), outpatient, inpatient (medical and surgical), emergency, psychiatry, TB clinic, youth-friendly or counseling wards. It has 861 employees (male 411, female 450) with 61 general physicians, 14 specialists, 178 nurses, 47 midwifery, 38 pharmacy, 22 health officers, 9 anesthesia, 1 dentist, 35 laboratory, 7 psychiatry professionals, 5 radiographers, and 444 supportive staff" might be omitted.

2. Why have the researchers chosen the time period "January 1st, 2016, to December 31st, 2020"?

3. The study design lacks clarity. Consider adopting a retrospective cohort study design based on nutritional status exposure. This design could be more suitable for addressing the research question.

4. The writing styles should be in manuscript form. The researcher can elaborate on the participants and the eligibility criteria. The source and study population can be written within a single paragraph.

5. It would be valuable to include a diagram illustrating the screening process, depicting the number of participants screened and reasons for exclusion.

6. The listed variables, research title, and research questions are not consistent. Initially, I anticipated nutritional status as the exposure and TB treatment outcomes as the outcome variable, assuming a retrospective cohort study design.

7. The data collection period is repeated in the study setting and period as well as the data collection section. It appears that the researchers may not have thoroughly reviewed the manuscript.

8. For the purpose of replication, it is important for the researchers to provide a citation for the development of the checklist. Additionally, consider uploading the checklist as a supplementary file.

9. The terms "successful" and "unsuccessful" treatment outcomes should be explicitly defined.
