# Supplementary material for: Nutritional status and treatment outcomes of tuberculosis in Mizan Tepi University Teaching Hospital, a five -year retrospective study
Source: PLoS One. 2024 Feb 15;19(2):e0298244. doi: 10.1371/journal.pone.0298244 (PMC10868852; doi:10.1371/journal.pone.0298244)
Supplement: Attachment — Submitted filename: Response to Reviewers.docx [file pone.0298244.s004.docx]

**Date. 01/12/2024**

**Response to Editor and Reviewers**

Dear editor, this is the response letter for our manuscript titled “Nutritional Status and Treatment Outcomes of Tuberculosis in Mizan-Tepi University Teaching Hospital - A five year Retrospective Study”. All of the comments are addressed carefully as follows.

**Response to Editor Comments:**

**Editor Comment 1**: Please ensure that your manuscript meets PLOS ONE's style requirements, including those for file naming.

**Response 1:** Thank you for your comment**.** We have accepted your comment and correction has made accordingly.

**Editor Comment 2:** We suggest you thoroughly copyedit your manuscript for language usage, spelling, and grammar. If you do not know anyone who can help you do this, you may wish to consider employing a professional scientific editing service.

**Response 2:** Thank you for your comment**.** We have accepted your comment and correction has made accordingly.

**Editor Comment 3:** We noticed you have some minor occurrence of overlapping text with the following previous publication(s), which needs to be addressed: In your revision ensure you cite all your sources (including your own works), and quote or rephrase any duplicated text outside the methods section. Further consideration is dependent on these concerns being addressed.

**Response 3:** Thank you for your comment**.** We have accepted your comment and correction has made accordingly.

**Editor Comment 4:** Please amend either the title on the online submission form (via Edit Submission) or the title in the manuscript so that they are identical.

**Response 4:** Thank you for your comment**.** We have accepted your comment and correction has made accordingly.

**Editor Comment 5:** Please amend either the abstract on the online submission form (via Edit Submission) or the abstract in the manuscript so that they are identical.

**Response 5:** Thank you for the comment**.** We have accepted your comment and correction has made accordingly.

**Editor Comment 6:** We note you have included a table to which you do not refer in the text of your manuscript. Please ensure that you refer to Table 2 in your text; if accepted, production will need this reference to link the reader to the Table.

**Response 6:** Thank you for your comment**.** We have accepted your comment and correction has made accordingly.

**Editor Comment 7:**  Please include captions for your Supporting Information files at the end of your manuscript, and update any in-text citations to match accordingly.

**Response 7:** Thank you for your comment**.** We have accepted your comment and correction has made accordingly.

**Editor Comment 8:**  Please review your reference list to ensure that it is complete and correct. If you have cited papers that have been retracted, please include the rationale for doing so in the manuscript text, or remove these references and replace them with relevant current references. Any changes to the reference list should be mentioned in the rebuttal letter that accompanies your revised manuscript. If you need to cite a retracted article, indicate the article’s retracted status in the References list and also include a citation and full reference for the retraction notice

**Response 8:** Thank you for your comment**.** We have accepted your comment and we have reviewed our reference list.

**Response to Reviewers' comments**

**Reviewer #1**

**Reviewer #1 general comment**: The research title is highly relevant and scientifically significant. However, the research design and the approach to conducting the study may not be appropriate for effectively addressing the research questions. Additionally, the manuscript's writing style appears outdated and does not align with modern standards. Significant revisions are required for the manuscript. Furthermore, the English writing in the manuscript could benefit from substantial improvement.

**Response to the general comment:** We thank the reviewer for the constructive feedback. We have accepted your comment and revision has made accordingly.

**Reviewer #1comment on abstract**

**Comment 1 on abstract background**: The research gap that the current study intends to address is not well described. The researcher states that there is limited information regarding the impact of nutritional status on TB treatment outcomes. However, there have been studies investigating the association between nutritional status and TB treatment outcomes. I suggest revising the background section to focus on the relationship between nutritional status and TB treatment outcomes, clearly highlighting the existing research gaps.

**Response 1 on abstract background:** Thank you for your comment**.** We have accepted your comment and correction has made accordingly.

**Comment 2 on abstract method:** The study design employed by the researchers is not clearly articulated. Was it a retrospective cohort study? If it was indeed a retrospective cohort study, they need to define the exposure and the outcome. Additionally, they should describe the timing of nutritional status assessment and treatment outcomes. Was the nutritional status evaluated at the initiation of treatment, after the second month of treatment, or after six months of treatment?

**Response 2 on abstract method:** Thank you for your comment. The study design employed was a retrospective cross-sectional study. Nutritional status was determined by the body mass index (BMI) that was measured during the initiation of anti-TB treatment.

**Comment 3 on abstract result:** The statement "A total of 625 TB patients with complete information were included from the registration book" could be relocated to the methods section. The results presented in the abstract should be aligned with the main objective of the research. Other findings may be included in the results section if they are deemed pertinent. This section requires further revision.

**Response 3 on abstract result:** Thank you for your comment. We have accepted your comment, and corrections have been made accordingly. Our main objective was to evaluate the effect of nutritional status along with other factors on TB treatment outcomes. We believe that including findings like descriptive statistics could be beneficial for readers in addition to the main findings, which is why we include other findings.

**Comment 4 on abstract conclusion:** The conclusion section also needs to be aligned with the research objectives and major findings. The primary aim of the research was not to determine the magnitude of treatment success.

**Response 4 on abstract conclusion:** Thank you for your comment. We have accepted your comment, and corrections have been made accordingly.

**Reviewer #1comment on introduction**

**Comment 1:** It is advisable to merge the first and second paragraphs. Summarizing the burden of TB and its related mortality in a single paragraph would be more effective. This approach would allow the researchers to discuss the relationship between nutritional status and TB treatment outcomes more comprehensively within the introduction section.

**Response 1:** Thank you for your comment. We have accepted your comment, and corrections have been made accordingly.

**Comment 2:** Other factors associated with TB treatment outcomes fall beyond the scope of this research, given the misalignment between the researchers' title and objective.

**Response 2:** Thank you for your comment. Examining the effect of other factors, along with nutritional status, on TB treatment outcomes is part of the objective of this research.

**Comment 3:** The researchers have omitted mention of existing studies investigating the association between nutritional status and TB treatment in Ethiopia within the introduction section. While they have referenced other studies focusing on factors affecting TB treatment outcomes in Ethiopia, this omission suggests a bias in that section.

**Response 3**: Thank you for your comment. We have accepted your comment, and corrections have been made accordingly.

**Reviewer #1comment on methods**

**Comment 1:** Study area and period: Some of the information provided appears unnecessary for the study. The description of the setting is crucial for replication and generalization. It's advisable to include only pertinent and relevant information. For instance, the statement "The hospital has antenatal care (ANC), outpatient, inpatient (medical and surgical), emergency, psychiatry, TB clinic, youth-friendly or counseling wards. It has 861 employees (male 411, female 450) with 61 general physicians, 14 specialists, 178 nurses, 47 midwifery, 38 pharmacy, 22 health officers, 9 anesthesia, 1 dentist, 35 laboratory, 7 psychiatry professionals, 5 radiographers, and 444 supportive staff" might be omitted.

**Response 1:** Thank you for your comment. We have accepted your comment, and corrections have been made accordingly.

**Comment 2:** Why have the researchers chosen the time period "January 1st, 2016, to December 31st, 2020"?

**Response 2:** Thank you for your comment. We chose this time period because organized registration of variables, including nutritional status, started on January 1^st^, 2016 at Mizan Tepi University Teaching Hospital. In addition, we aimed to see the effect of multiple factors on TB treatment outcomes in the study area by using the most recent five-year registration data.

**Comment 3:** The study design lacks clarity. Consider adopting a retrospective cohort study design based on nutritional status exposure. This design could be more suitable for addressing the research question.

**Response 3:** Thank you for your comment. Since we aimed to evaluate the effect of multiple factors in addition to nutritional status, we conducted a retrospective cross-sectional study design.

**Comment 4:** The writing styles should be in manuscript form. The researcher can elaborate on the participants and the eligibility criteria. The source and study population can be written within a single paragraph.

**Response 4:** Thank you for your comment. We have accepted your comment, and corrections have been made accordingly.

**Comment 5:** It would be valuable to include a diagram illustrating the screening process, depicting the number of participants screened and reasons for exclusion.

**Response 5:** Thank you for your comment. We have accepted your comment, and corrections have been made accordingly.

**Comment 6:** The listed variables, research title, and research questions are not consistent. Initially, I anticipated nutritional status as the exposure and TB treatment outcomes as the outcome variable, assuming a retrospective cohort study design.

**Response 6:** Thank you for your comment. Our research aimed to assess the effect of nutritional status along with other multiple factors on TB treatment outcomes.

**Comment 7:** The data collection period is repeated in the study setting and period as well as the data collection section. It appears that the researchers may not have thoroughly reviewed the manuscript.

**Response 7:** Thank you for your comment. Previously, the data collection period was included in the study setting and period section, but based on the previous comment from PLOS ONE, we included the data collection period as a separate section.

**Comment 8:** For the purpose of replication, it is important for the researchers to provide a citation for the development of the checklist. Additionally, consider uploading the checklist as a supplementary file.

**Response 8:** Thank you for your comment. We have accepted your comment, and corrections have been made accordingly.

**Comment 9:** The terms "successful" and "unsuccessful" treatment outcomes should be explicitly defined.

**Response 9:** Thank you for your comment. We have accepted your comment, and corrections have been made accordingly.

**Reviewer #2**

**Abstract**

**Comment 1**: The abstract successfully outlines the study's objectives and key findings. However, it lacks specific details about the methodology, such as the type of logistic regression analysis used. Include brief methodological details in the abstract for a more comprehensive overview.

**Response 1:** Thank you for your feedback. We have accepted your comment, and corrections have been made accordingly.

**Background**

**Comment 1:** The background provides a good context of TB's global and local impact, yet it could benefit from a more direct link to the study's focus on nutritional status.

Improvement Suggestion: Integrate recent studies or data showing the direct impact of nutritional status on TB outcomes to strengthen the rationale for this study.

**Response 1:** Thank you for your feedback. We have accepted your comment, and corrections have been made accordingly.

**Methods**

**Comment 1:** Feedback: The methods section is well-structured, but it lacks detail on the criteria for nutritional status assessment and how undernutrition was defined in the study. Moreover, the application of STROBE guidelines for observational studies is not clearly articulated.

Improvement Suggestion: Clarify the criteria and metrics used for evaluating nutritional status. Explicitly outline adherence to STROBE guidelines, emphasizing aspects like study design, setting, participants, variables, data sources/measurement, and statistical methods. Detailing these elements will enhance the study's methodological transparency and reproducibility.

**Response 1:** Thank you for your feedback. We have accepted your comment, and corrections have been made accordingly.

**Results**

**Comment 1:** The results are clearly presented, but the connection between nutritional status and TB treatment outcomes needs more emphasis.

Improvement Suggestion: Provide a more detailed analysis of how nutritional status specifically influenced treatment outcomes, including breakdowns of data according to different nutritional categories.

**Response 1**: Thank you for your feedback. We tried to show how nutritional status influenced treatment outcomes in Table 3. As it is presented in Table 3, about 32.4% (62/191) of undernourished TB cases had poor treatment outcomes, while only about 21.3% (83/389) of normal-weight TB cases had poor treatment outcomes.

**Conclusion**

**Comment 1**: Feedback: The conclusion summarizes the findings well. However, it does not sufficiently address the broader implications of the study.

Improvement Suggestion: Expand the conclusion to include potential impacts on public health policies and future research directions, especially focusing on nutritional interventions in TB management.

**Response 1:** Thank you for your feedback. We have accepted your comment, and corrections have been made accordingly.

**General Observations**

**Comment 1**: Coherence and Structure: The manuscript maintains a logical flow. However, the connection between sections could be stronger, particularly between the background and the study's focus on nutrition.

**Response1**: Thank you for your feedback and we have accepted your comment.

**Comment 2: Alignment of Sections:** Each section aligns well with the overall objective, but the link between nutritional status and TB outcomes could be more pronounced throughout the manuscript.

**Response2**: Thank you for your feedback and we have accepted your comment.

**Comment 3:** Critical Analytic Feedback: The study presents significant findings but lacks depth in exploring the mechanisms by which nutritional status affects TB outcomes.

**Response 3:** Thank you for your feedback and we have accepted your comment.

**Comment 4:** Missing Information: The study does not address potential confounding factors such as socioeconomic status or access to health care, which could influence both nutritional status and TB outcomes.

**Response 4:** Thank you for your feedback and we have accepted your comment. We tried to discuss the missing information as a limitation in our study.
